# Typhoid Fever: Tracking the Trend in Nigeria

**DOI:** 10.4269/ajtmh.18-0045

**Published:** 2018-07-25

**Authors:** Kabiru Olusegun Akinyemi, Akeeb Oriowo Bola Oyefolu, Wasiu Bamidele Mutiu, Bamidele Abiodun Iwalokun, Edward Sunday Ayeni, Samuel Oluwasegun Ajose, Stephen K. Obaro

**Affiliations:** 1 Department of Microbiology, Lagos State University, Lagos, Nigeria;; 2 Department of Medical Microbiology and Parasitology, College of Medicine, Lagos State University, Lagos, Nigeria;; 3 Biochemistry Unit and Malaria Research Laboratory, Nigerian Institute of Medical Research, Lagos, Nigeria;; 4 Records Unit, Lagos State University Teaching Hospital, Lagos, Nigeria;; 5 Division of Pediatric Infectious Disease, University of Nebraska Medical Centre, Omaha, Nebraska;; 6 International Foundation Against Infectious Diseases in Nigeria (IFAIN), Abuja, Nigeria

## Abstract

Typhoid fever continues to pose a serious health challenge in developing countries. A reliable database on positive blood cultures is essential for prompt interventions. To generate reliable data on *Salmonella enterica* serovar Typhi (*S.* Typhi)–positive blood culture trends in typhoidal *Salmonella* in Nigeria alongside changing contextual factors and antimicrobial resistance patterns, a retrospective cohort study was conducted in two hospitals in Lagos between 1993 and 2015. Medical records of typhoid patients were reviewed for positive culture and antibiogram, using standard procedures and analyzed. Additional data were retrieved from a previous study in seven facilities in Abuja and three hospitals in Kano from 2008 to 2017 and 2013 to 2017, respectively. A declining trend in percent positivity of *S.* Typhi was observed in Abuja with more erratic trends in Lagos and Kano. In Lagos, more than 80% of the isolates from the entire study period exhibited multiple drug resistance with a generally increasing trend. Of the chosen contextual factors, improvements were recorded in female literacy, access to improved water supply, diarrheal mortality in children younger than 5 years, gross domestic product, and poverty while access to improved sanitation facilities decreased over time nationally. Typhoid fever still poses a serious health challenge in Nigeria and in antibiotic resistance, and is a major health security issue. A combined approach that includes the use of typhoid vaccines, improvements in sanitation, and safe water supply is essential.

## INTRODUCTION

In 2010, the global estimate of typhoid fever caused by *Salmonella enterica* serovar Typhi (*S.* Typhi) was estimated to be 26.9 million cases with 217,000 deaths recorded. This estimate was adjusted for blood culture sensitivity based on a conservative assumption of 50%.^1,2^ However, only Egypt and South Africa contributed to this estimate for the African continent. A previous global estimate of the burden of typhoid fever indicated that south-central and east-central Asia had the highest incidences of typhoid fever with more than 100 cases per 100,000 people annually; Africa was estimated to have a medium incidence (10–100 cases per 100,000).^3^ The estimated number of typhoid fever cases in low- and middle-income countries in 2010 after adjusting for water-related risk was 11.9 million (95% confidence interval: 9.9–14.7) cases with 129,000 (75,000–208,000) deaths.^4^ It is clear that the incidence of typhoid fever in Africa is still not yet well understood.^5^ Out-of-sample validation of the model against data from nine Typhoid Fever Surveillance in Africa Program sites showed that the model has mixed success in predicting incidence for locations outside the estimation sample.^5^

The paucity of epidemiological data regarding invasive *Salmonella* disease in sub-Saharan Africa led the World Health Organization (WHO) to call for a continent-wide approach in generating more accurate disease incidence and antimicrobial susceptibility data in 2008.^6^ In Nigeria, typhoid fever remains a major disease because of factors such as increased urbanization, inadequate supplies of potable water, regional movement of large numbers of immigrant workers, inadequate facilities for processing human waste, overburdened health-care delivery systems, and overuse use of antibiotics that contribute to the development and spread of antibiotic-resistant *S.* Typhi.^7,8^ However, the true incidence of typhoid fever is difficult to evaluate in Nigeria because of the lack of a proper coordinated epidemiological surveillance system. Nevertheless, information on typhoid fever prevalence has been documented by several researchers in some states in Nigeria ranging from 0.071% in Oyo to 47.1% in Osun.^8–15^

Blood culture–positive typhoidal *Salmonella* remains the pivotal determinant to estimate true burden. Unfortunately, only few hospitals, specifically, referral hospitals, perform blood culture for diagnosing typhoid cases. The rate of hospitalization and prolonged illness of patients with typhoid fever in high-burden regions due to treatment failure with empirical therapy is a continuing public health concern.^16^ Since the early 1990s, the spread of multiple drug–resistant (MDR) *S.* Typhi strains (resistant to first-line drugs: ampicillin, chloramphenicol, and trimethoprim–sulfamethoxazole), and more recently, ciprofloxacin have been observed in parts of Asia and Africa, making the treatment of typhoid fever more challenging.^16^ Over the years, a similar resistance pattern was observed in Nigeria.^17–19^ Currently, the two internationally licensed typhoid vaccines^20^ have not yet been considered for incorporation into the Expanded Immunisation Program of health policy in Nigeria. An additional barrier to reducing typhoid fever incidence in Nigeria is the lack of access to safe drinking water and improved sanitation facilities.^21^ The present study was undertaken to generate comprehensive and reliable data on *S.* Typhi blood culture positivity with a view to outline the longitudinal trends of typhoid fever in parallel with key contextual factors and to assess the trends of antimicrobial resistance in *S.* Typhi in Nigeria.

## METHODS

### Study setting and population.

Nigeria is the most populated country in sub-Saharan Africa with a population of about 187 million in 2015^22^ with Lagos State, Abuja, and Kano making up 11% of the population at approximately 20 million people in 2016.^23^ A retrospective cohort study on blood culture–positive typhoid fever was conducted in two referral public hospitals in Lagos: Lagos State University Teaching Hospital, Ikeja, and General Hospital Lagos (GHL) between 1993 and 2015. Medical records of clinically diagnosed and blood culture–confirmed patients with typhoid fever were reviewed for these periods. In addition, blood culture–confirmed data from children younger than 5 years of age in three facilities in Kano and seven facilities in Abuja were analyzed alongside the data from Lagos (Table 1).^15^ All data included in this study were anonymized.

**Table 1:** Characteristics of study hospitals

State	State level population^24^*	City/region	Hospital	Payment required for health care
Lagos	9,113,605	Ikeja	Lagos State University Teaching Hospital	Yes
		Lagos	General Hospital Lagos	Yes
Kano	9,401,288	Tarauni, Kano	Aminu Kano Teaching Hospital	Yes
		Kano city	Hasiya Bayero Pediatric Hospital	Yes
		Kano city	Murtala Specialist Hospital	Yes
Abuja	1,406,239	Abuja	The National Hospital, Abuja	Yes
		Gwagwalada	University of Abuja Teaching Hospital	Yes
		Nyanya District	Nyanya District Hospital	Yes
			Zankli Medical Center	Yes
		Garki	Garki General Hospital	Yes
		Maitama district 5	Maitama Hospital	Yes
		Keffi	Federal Medical Center Keffi	Yes

* Population data based on 2006 census.

### Sources of contextual factors.

The patients did not pay for diagnostic services in facilities in Lagos and Ikeja.

The national contextual factor data used in this study were obtained from standard sources. Data on improved sanitation and improved water sources, and female literacy were obtained from the WHO/United Nation International Children Emergency Fund Joint Monitoring Programs World Bank^25^ and United Nations Educational Scientific and Cultural Organization,^26^ respectively. National poverty data were obtained from World Bank Global Poverty Working Group.^27^ Data on real gross domestic product (GDP) were from International Monetary Fund World Economic Outlook.^28^ Diarrheal mortality was extracted from a 2014 Lancet review on cause-specific under-five mortality from the Child Health Epidemiology Reference Group.^29^

### Ethical permission.

Approval from the Lagos State Health Service Commission was sought and granted. For the Kano and Abuja sites, ethical approval was sought from each of the facility review boards, including the Federal Capital Territory of Nigeria Ethics Committee, Zankli Medical Center Ethics Committee, and National Hospital Abuja Ethics Committee among many others.^30^

### Case definition, data collection, and inclusion criteria.

In Lagos, typhoid fever cases were defined in this study as patients presenting with fever lasting for more than 3–5 days with one or more of the following symptoms: diarrhea, vomiting, loss of appetite, persistent headache, malaise etc., as noted by the physicians and the total *S.* Typhi–positive cultures in each hospital out of all cultures performed in a given year. Patient information data such as age, gender, underlying health conditions, deaths, and antibiotic resistance were obtained from the records. All cases were assumed to be domestically acquired.

The inclusion criteria for case selection at the sites in Kano and Abuja are described in detail in a previous publication.^30^ In Abuja, seven hospitals were included: 1) The National Hospital, Abuja, 2) University of Abuja Teaching Hospital, Gwagwalda, 3) Nyanya District Hospital, 4) Zankli Medical Center, 5) Garki General Hospital, 6) Maitama Hospital, and 7) Federal Medical Center, Keffi. Three sites were included in Kano: 1) Aminu Kano Teaching Hospital, 2) Hasiya Bayero Pediatric Hospital, and 3) Murtala Specialist Hospital.^30^ Consent was solicited from parents or guardians of children younger than 5 years presenting with fever lasting more than 3 days along with additional symptoms, including diarrhea and convulsions, as noted by a nurse in the triage department.^30^

### Microbiological procedures and susceptibility testing.

In Lagos, laboratory diagnosis for suspected typhoid cases were first based on Widal agglutination tests with a cut-off titer of 1:160 for O and H agglutinins. Only Widal-positive blood was considered for blood culture as a practice in public facilities in Lagos, except in cases of complications. The blood cultures were performed according to standard procedures.^31^ Antimicrobial susceptibility tests in Lagos were determined by the agar diffusion method.^32^ To determine antimicrobial susceptibility in Kano and Abuja, the Epsilometer test (EtestbioMérieux) was performed in accordance with standard methodology. The minimum inhibitory concentration results were analyzed using the standards established by the Clinical and Laboratory Standards Institute.^15^

Analysis of data: Simple descriptive statistics were used to assess trends. We computed the number of *S.* Typhi–positive cultures out of the total number of blood cultures performed to yield a percent positivity of typhoid as a subset of tabulated by time to describe the temporal trends of typhoid in the dataset. We chose not to pool data from different sites because we judged that the geographical heterogeneity was of primary interest.

## RESULTS

### Trend and case fatality rate of typhoid fever.

**Figure 1.**
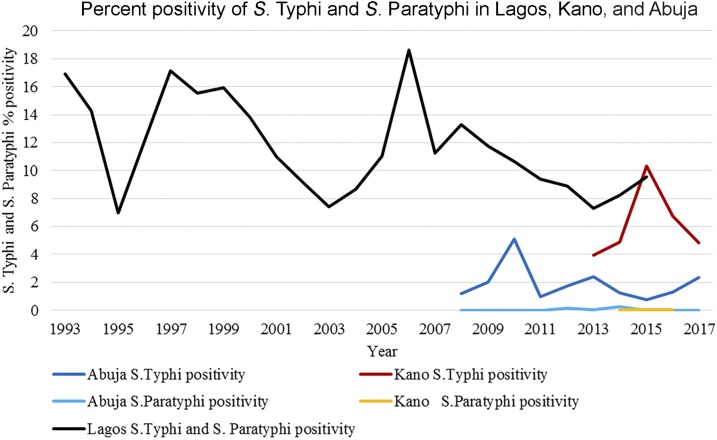
Percent positivity of *Salmonella enterica* serovar Typhi (*S.* Typhi) and *S.* Paratyphi in Lagos, Kano, and Abuja. The dark blue line shows the *S.* Typhi percent positivity in Abuja. The light blue line shows the *S.* Paratyphi percent positivity in Abuja. The black line shows the *S.* Typhi and *S.* Paratyphi percent positivity in Lagos. The red line shows the *S.* Typhi percent positivity in Kano. The yellow line shows the *S.* Paratyphi percent positivity in Kano.

The number of cultures performed and availability of data across Lagos, Kano, and Abuja varied (Table 2). In Lagos, *S.* Typhi percent positivity ranged between 7% and 18.6% over a 23-year time period. There was no clear trend in *S.* Typhi isolation over the time period and rates have remained less than 10% since 2010 (Figure 1). The highest positivity rates were recorded in 1997 and 2006 at 17.1% and 18.6%, respectively. Serotyping of isolates was not performed in Lagos and the culture positivity rates included both *S.* Typhi and *S.* Paratyphi isolates. The Kano and Abuja sites measured the trend as the number of *S.* Typhi–positive cultures out of the total number of blood cultures performed to yield a percent positivity trend. Within the period of 2013–2017, the blood culture–confirmed typhoid incidence in Kano ranged from 3.9% to 10.4%. Similarly, in Abuja, the percent positivity of *S.* Typhi ranged from 0.8% to 2.4% across the period of 2008–2017. Abuja showed a generally decreasing trend with a slight increase between 2015 and 2017, whereas no clear trend was evident in Kano. *S.* Paratyphi culture positivity remained consistently low in both Kano and Abuja at less than 1% throughout the study period.

**Table 2:** Study site characteristics

Study site	Period	Total *S.* Typhi/Paratyphi A–positive cultures
Abuja	2008–2017	*S.* Typhi: 230
		*S.* Paratyphi A: 10
Kano	2013–2017	*S.* Typhi: 920
		*S.* Paratyphi A: 4
Lagos	1993–2015	Enteric fever: 440

*S*. Typhi = *Salmonella enterica* serovar Typhi.

### Antimicrobial resistance in *S.* Typhi.

**Figure 2.**
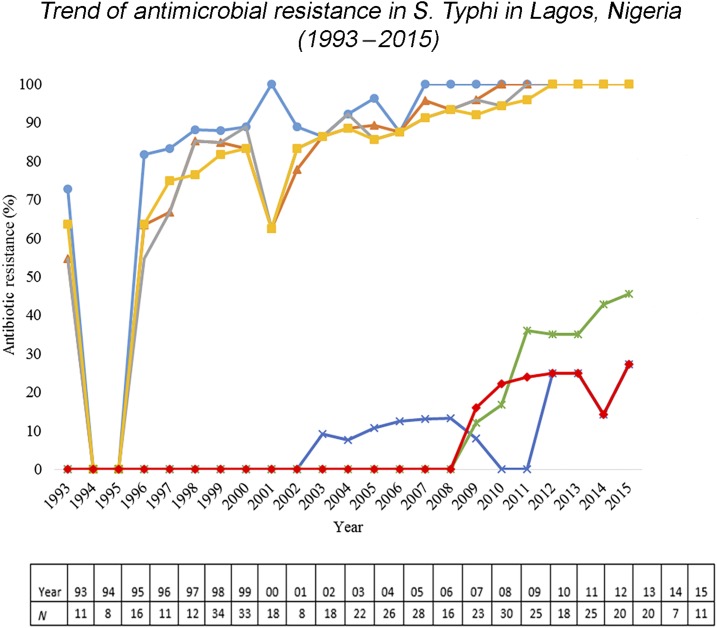
Trend of antimicrobial resistance in *Salmonella enterica* serovar Typhi (*S.* Typhi) in Lagos, Nigeria (1993–2015). Antimicrobial resistance trends in Lagos from 1993 to 2015. The light blue line shows the percentage of ampicillin-resistant *S.* Typhi. The orange line shows the percentage of chlora6mphenicol-resistant *S.* Typhi. The gray line shows the percentage of co-trimoxazole-resistant *S.* Typhi. The yellow line shows the percentage of tetracycline-resistant *S.* Typhi. The dark blue line shows the percentage of ciprofloxacin-resistant *S.* Typhi. The green line shows the percentage of cefuroxime-resistant *S.* Typhi. Cefuroxime was introduced as of 2003. The red line shows the percentage of cefotaxime-resistant *S.* Typhi. Cefotaxime was introduced as of 2008.

More than 80% of the 440 *S.* Typhi isolates from Lagos were MDR. A general increase in the trend of MDR *S.* Typhi was observed across the study time period (Figure 2). There was an increase in ampicillin resistance in *S.* Typhi from 81.8% in 1996 to 100% in 2008 which remained constant through 2015 with similar increases for chloramphenicol (63.6–100%), co-trimoxazole (54.6–100%), and tetracycline (63.6–100%) over the same time period (Figure 2). The emergence of resistance in *S.* Typhi to ciprofloxacin was first observed in 2003. Nine percent of the 22 *S.* Typhi isolates in this year were found to be resistant. A rapid increase in cefotaxime-resistant *S.* Typhi from 16.0% in 2009 to 25% in 2013 was observed with a peak of 27.3% in 2015. Similarly, cefuroxime-resistant *S.* Typhi increased steadily from 12% in 2009 to 45.5% in 2015. Antimicrobial resistance trends from Abuja were unavailable as only 9 months of data were available from these sites.^15^ Aggregated resistance data were, however, reported for the study period. *S.* Typhi isolated from Abuja showed 0% resistance to ceftazidime, ofloxacin, ciprofloxacin, and ceftriaxone, whereas just less than 50% of isolates were resistant to amoxicillin, augmentin, and chloramphenicol. In Kano, antimicrobial resistance results were previously described.^15^ However, disaggregated data by year were not presented; thus, clear trends in resistance cannot be ascertained.

### Typhoid fever and contextual factors.

**Figure 3.**
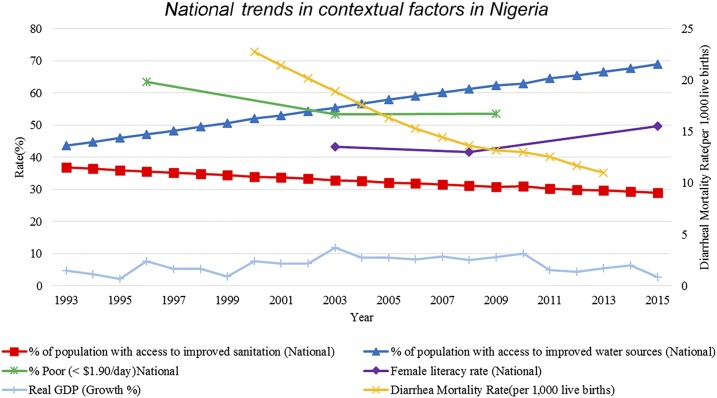
National trends in contextual factors in Nigeria. This figure shows a series of typhoid-relevant contextual factors. The red line marked with squares shows the percentage of the population in Nigeria with access to improved sanitation facilities. The green line marked with stars shows the percentage of the population in Nigeria living on less than $1.90/day. The light blue line marked with hash lines shows the percentage of real Gross Domestic Products (GDP) growth. The dark blue line marked with triangles shows the percentage of the population in Nigeria with access to improved water sources. The purple line marked with diamonds shows the percentage of female literacy in Nigeria. The yellow line marked with x’s shows the diarrheal mortality rate per 1,000 live births in Nigeria.

There was a steady decreasing trend in the level of improved sanitation in both rural and urban settings from 1993 to 2015 with a simultaneous increase in access to improved water supply (Figure 3). Trends related to both improved sanitation facilities and improved water supply are measured as access to improved sanitation and water out of the urban and rural populations, respectively, collected from census data and nationally representative survey data at the household level.^25^ Diarrheal mortality in children younger than 5 years also exhibited a sharp decline nationally from 23 per 1,000 live births in 2000 to 11 per 1,000 live births in 2013. There was a decrease in the poverty rate (people who live on less than $1.90 per day) from 1992 to 2009, in spite of inflation and devaluation of local currency (Naira) and an increase in female literacy from 2008 to 2015. The real GDP growth from 1993 to 2015 for Nigeria showed a generally increasing trend during the study time period.

## DISCUSSION

The percent positivity trends from the Lagos, Abuja, and Kano show considerable variation with clear declines over time only evident in Abuja. This highlights the regional variation in typhoid burden in Nigeria and the significant gaps in surveillance. Other countries in the region also exhibit significant variation in typhoid incidence. In Burkina Faso, the annual incidence of typhoid fever has been reported to be between 107 and 402 cases per 100,000 persons in Nikoko and Polesgo, respectively.^33^ In Libya, the incidence rate for the year 2006 was 16 per 100,000 persons per year,^34^ and Egypt remains a country with incidence rates between 1 and 180 per 100,000 cases of enteric fever over a 7-year period.^35^

The reasons that the percent of blood cultures positive for typhoid varies in this study may be attributed to factors such as geographical location, cultural beliefs, and/or socioeconomic considerations that limit the population’s access to laboratory tests. Operational factors, such as changes in laboratory capacity, the medical guidelines, and/or protocols, and budgetary restraints for blood culture could also be responsible. For example, underestimation bias from the use of only Widal-positive blood samples for cultures in most of the facilities due to the limited resources might make it possible to screen out positive typhoid. Also, blood cultures were carried out manually, and thus, might have affected the sensitivity of culture results. Similar operational factors and many more had been observed and reported to affect typhoid fever incidence in LIMCs.^36^ Also, an automated blood culture apparatus such as BacTec or BacT/ALERT (Artisan Technology Group, Champaign, IL) usually enhances the sensitivity of the culture to detect *S.* Typhi.^5^

In Lagos, we recorded a higher trend of *S.* Typhi–infected individuals in 1998, 1999, 2005, and 2008. In Kano, the peak trends of culture-positive *S.* Typhi were observed in 2010, 2013, and 2017. The highest isolation of *S.* Typhi–positive cultures in Abuja occurred in 2015. These peaks could in part be explained by decreased access to improved sanitation facilities and the quality of water. Studies have documented nonconformity of well- and tap water with WHO standards and guidelines of National Agencies for Food and Drug Administration Control in Nigeria with the increases in typhoid fever in Lagos being attributed to this.^37,38^

Resource and financial limitations are additional factors that account for the poor quality of water, sanitation, and hygiene infrastructure in Nigeria. There is a lack of political will on the part of water regulatory authorities such as States Water Corporations and Federal Municipal Water Agencies to monitor pipe water leakages and enforcement of existing laws on quality of water supply. Contamination of public water supplies from the few functional government water plants is still a common experience because of public vandals of water pipes, leakage of old unrepaired pipes, and particles from the water corporation.^37^ In Lagos specifically, increasing urbanization has led to government interventions having a marginal impact on the vehicles of disease transmission. The recent deployment of environmental health officers to monitor the sanitary conditions of every household by the Lagos State government is an intervention geared toward reduction of typhoid fever and other infectious diseases. This proactive step on the part of the government might help explain the decline in the observed cases of typhoid fever in the last 2 years of this study. There was a steady decreasing trend in the level of improved sanitation in both rural and urban settings in Nigeria which might have contributed to a high typhoid fever trend. In Lagos, the estimated population increased from 7,268,199 in 1993 to 20,655,392 in 2015, with about 10,000 metric tons of waste generated daily,^39^ which in turn hinders the proper functioning of sewage systems.

The antimicrobial resistance trend from Lagos is based on a small sample size with overall MDR showing a gradual increase from 70% in 1996 to 100% in 2015.

These findings are consistent with a recent report from Northern Nigeria, where more than 60% of *S.* Typhi isolates were MDR.^15^ Similar results have been observed in a number of African countries such as Kenya,^40^ Tanzania, Burkina Faso, Guinea Bissau, and Madagascar.^41^ Low MDR and/or reduced fluoroquinolone susceptibility has also been documented in West African countries, such as Senegal and Ghana.^41,42^ Our findings are also consistent with MDR *S.* Typhi isolates reported in several sites in sub-Saharan Africa, and reduced susceptibility to ciprofloxacin identified in *S.* Typhi from Kibera, Kenya, and Pietermaritzburg, South Africa in recent time.^3^

The high prevalence of antimicrobial-resistant *S.* Typhi recorded in Lagos may be attributed to nonprescription antibiotic use, over-the-counter sale of antibiotics, and nonadherence to the prescribed dose of antibiotics. The documented sale of fake, adulterated, or outdated antibiotics at unregulated pharmacies has made it difficult to establish the true prevalence of antibiotic consumption in Nigeria.^43^ The emerging resistance trends imply that drugs that formed the main therapy for typhoid patients in the early 1990s and 2000s are no longer effective.

### Limitations.

The true burden of typhoid is likely to be underreported because ill individuals do not always seek health care at hospitals. Furthermore, given the time required to confirm bacteremia microbiologically, patients with febrile symptoms are usually given antibiotics before laboratory confirmation of typhoid, which may have influenced the present culture data. The contention that antibiotics lower the sensitivity of blood cultures had been documented in studies elsewhere.^44,45^

The data from Lagos are based on records of the two referral hospitals which represent less than 10% of the public health hospitals in Lagos. In addition, laboratory services for confirmation of cases are not free. Similarly, the data from Kano and Abuja do not accurately represent the populations of their respective areas as only children younger than 5 years were included. These facilities enrolled children with the greatest illness severity and had limited beds available for admission, as such the data collected underrepresents the true trend of typhoid fever in children younger than 5 years. As a result, the burden of typhoid fever estimated in this study might be considerably lower than the true burden in Lagos, Abuja, and Kano. Loss of data to fires in GHL, unavailability of some data in the medical records because of strike action, irregular record keeping observed in these health facilities and inability to uniformly record data on patient blood volumes drawn could affect the overall representation of the results as well.^15,36^

Paratyphoid fever data were unavailable in Lagos and limited in both Abuja and Kano. Enteric fever trend data from certain South Asian countries have shown an increasing trend in paratyphoid fever incidence in conjunction with decreasing levels of typhoid fever.^46^ The lack of data on paratyphoid fever is an important gap that should be addressed. The culture positivity data used in this study are subnational, whereas the contextual data used are nationally representative, making it difficult to ascribe changes in typhoid occurrence to improving or deteriorating contextual factors. Last, all data in this study came from health facilities where it is probable that only the more severe and advanced cases of typhoid and paratyphoid fever are being captured. National surveillance programs or disease notification systems are likely to provide a more accurate estimate of burden and highlight subnational discrepancies.

## CONCLUSION

The data from these health facilities provided a unique tool to understand the epidemiology of typhoid fever in Nigeria. This study emphasizes the lack of reliable and consistent incidence data in Nigeria while also highlighting the importance of antibiotic resistance as a major health security issue. Typhoid fever still contributes significantly to the disease burden in the region, and it is imperative that more resources be dedicated toward tackling this disease. The data reported here can guide local health policy makers and international communities to develop strategic interventions aimed at controlling the spread of typhoid fever. A combined approach strategy that would include the use of typhoid vaccines and improvements in sanitation and safe water supply is essential. The government should increase the allocation of funds to the health sector and develop concerted strategies for monitoring infectious diseases and promote awareness of the availability of various health services to the general population.
